# The efficacy and safety of apatinib treatment for patients with advanced or recurrent biliary tract cancer: a retrospective study

**DOI:** 10.1186/s12885-021-07907-4

**Published:** 2021-02-23

**Authors:** Caiyun Nie, Huifang Lv, Yishu Xing, Beibei Chen, Weifeng Xu, Jianzheng Wang, Xiaobing Chen

**Affiliations:** 1 Department of Oncology, The Affiliated Cancer Hospital of Zhengzhou University, No. 127 Dongming Road, Jinshui District, Zhengzhou City, 450008 Henan Province China; 2 Henan Cancer Hospital, Zhengzhou, 450008 China

**Keywords:** Biliary tract cancer, Apatinib, Targeted therapy, Efficacy

## Abstract

**Introduction:**

The present study aims to evaluate the efficacy and safety of apatinib monotherapy or combination therapy for patients with advanced or recurrent biliary tract cancer (BTC).

**Methods:**

Twenty-eight patients with advanced or recurrent BTC who progressed after prior systemic therapies and treated with apatinib from January 2017 to June 2019 were enrolled in this retrospective and observational study. The primary end point was progression free survival (PFS). Secondary end points included overall survival (OS), objective response rate (ORR), disease control rate (DCR), and toxicity.

**Results:**

A total of 28 patients with advanced or recurrent BTC who progressed after prior systemic therapies received apatinib monotherapy or combination therapy (with capecitabine, S-1, oxaliplatin, irinotecan or PD-1 inhibitor), including 9 cases of gallbladder cancer and 19 cases of cholangiocarcinoma. Six patients achieved PR, 15 patients had SD and 7 patients had PD. Median progression-free survival (PFS) and overall survival (OS) was 4.3 months (95%CI = 1.8–6.8) and 6.2 months (95% CI = 4.6–7.8) respectively. The ORR and DCR were 21.4% (6/28) and 75.0% (21/28), respectively. Most of the adverse events were grade 1–2 in severity, apatinib treatment was well tolerated.

**Conclusions:**

Apatinib monotherapy or combination therapy can improve PFS in patients with advanced or recurrent BTC who progressed after prior systemic therapies, and adverse reactions can be well tolerated. Our study support apatinib therapy as a feasible therapeutic strategy in advanced or recurrent BTC.

**Supplementary Information:**

The online version contains supplementary material available at 10.1186/s12885-021-07907-4.

## Introduction

Biliary tract cancer (BTC) includes gallbladder cancers (GBCs) and cholangiocarcinomas (CCAs), further divided into intrahepatic cholangiocarcinomas (iCCAs) and extrahepatic cholangiocarcinomas (eCCAs). The incidence of iCCAs in the western world is rising, and the incidence of GBCs is on the decline [1, 2]. The onset of BTC is hidden, lacking specific clinical manifestations in the early stage, and most patients are already in the advanced stage at the time of diagnosis, losing the chance of radical surgical resection and having a poor prognosis. Advanced or recurrent BTC is dominated by systemic medications. Prospective randomized controlled studies have shown that systemic chemotherapy can prolong the survival of patients with BTC. However, the effect of systemic chemotherapy is limited. There is no effective second-line standard treatment after first-line chemotherapy. New treatment strategies are urgently needed [3, 4].

Apatinib is an oral, highly selective VEGFR2 antagonist, and was approved by the China Food and Drug Administration for use as a single agent in patients with advanced gastric or gastroesophageal junction adenocarcinoma after second line chemotherapy failure [5]. In addition, apatinib has also showed good clinical efficacy in a variety of solid tumors, including lung [6], liver [7], ovarian [8], breast [9], colorectal [10] and bone soft tissue sarcoma [11]. Preclinical studies demonstrated that apatinib affect VEGF-mediated cell proliferation and migration in cholangiocarcinoma cell [12], however, clinical use of apatinib in BTC was rarely investigated. This retrospective and observational study was performed to evaluate the efficacy and safety of apatinib monotherapy or combination therapy for patients with advanced or recurrent BTC who progressed after prior systemic therapies.

## Methods

### Patients

From January 2017 to June 2019, a total of 28 patients with advanced or recurrent BTC received apatinib monotherapy or combination therapy at the Affiliated Cancer Hospital of Zhengzhou University were enrolled in the present study. Inclusion criteria were pathologically confirmed as BTC, including gallbladder cancers and cholangiocarcinomas; advanced or recurrent BTC who progressed after prior systemic therapies according to imaging examination; have at least one measurable lesion. This study was carried out in accordance with the ethical guidelines of the 1975 Declaration of Helsinki and was approved by the ethics committee of the Affiliated Cancer Hospital of Zhengzhou University. Written informed consent was obtained from every patient for the use of the medical records for research purposes.

### Treatment

Apatinib was provided as tablets and administered orally daily until disease progression, unacceptable toxicity or death. In this study, the patients received two dosing regimens, including apatinib monotherapy or combination therapy. In the monotherapy regimen, apatinib was given at a dose of 500 mg once a day, and dose reduction to no less than 250 mg once a day was allowed. In the combination therapy regimen, apatinib was given at a dose of 250 mg once a day, and concurrent chemotherapy or immunotherapy was given simultaneously, including capecitabine, S-1, oxaliplatin, irinotecan or PD-1 inhibitor. There were no dose modifications but only delays or permanent discontinuation in this group.

### Efficacy and safety assessments

Imaging examination were performed after every 2 cycles to evaluate the efficacy of treatment. Clinical efficacy of patients was classified as having a complete response (CR), partial response (PR), stable disease (SD), or progressive disease (PD) according to RECIST version 1.1 response evaluation criteria in solid tumors. Objective response rate (ORR) was defined as the proportion of patients who achieved a confirmed CR or PR. Disease control rate (DCR) was defined as the proportion of patients who achieved CR, PR and SD. Adverse events (AEs) were assessed according to the Common Terminology Criteria for Adverse Events, version 4.0. During treatment, performance status, abdominal and cardiac ultrasound, electrocardiogram, complete blood count, liver and kidney function were monitored regularly in every cycle.

### Statistical analysis

Difference between groups were determined by Pearson’s chi squared test or Fisher’s exact test. Survival curves were estimated using the Kaplan-Meier method and the log-rank test was used to test for differences between the groups. PFS was defined as the period from the time of treatment with apatinib to disease progression or patient death due to any cause. OS was defined as the period from the time of treatment with apatinib to patient death from any cause or the last day of the follow-up. The follow-up deadline is December 31, 2019. All the statistical descriptive analyses were performed with SPSS 17.0 (SPSS, Chicago, IL) software.

## Results

### Patient and treatment characteristics

A total of 28 patients with advanced or recurrent BTC were included in this retrospective study. Patient and treatment characteristics are summarized in Table 1 and Table 2. The median age was 54 years (range 36–79), 12 female patients and 16 male patients. Nine patients were diagnosed as GBC and the other patients were CCA. Based on the anatomical locations, the 19 cases of CCA classified as 6 cases of intrahepatic, 8 cases of perihilar, and 5 cases of distal tumors. All the patients were advanced or recurrent BTC, common metastatic sites included intra-abdominal lymph node (57.1%), liver (50.0%), peritoneum (28.6%) and lung (28.6%) according to imaging examination. Other metastatic sites were mediastinum, supraclavicular lymph nodes, bones, and etc. All the patients in the study had undergone prior systemic therapies, so apatinib was given as second line treatment in 11 patients (39.3%) and third- or fourth-line treatment in 17 patients (60.7%). Twelve patients received apatinib as monotherapy and 16 patients received apatinib combination therapy.

**Table 1:** Patient and treatment characteristics

Characteristic	Total(*n* = 28) n (%)	Monotherapy(*n* = 12) n (%)	Combination therapy(*n* = 16) n (%)	*P*
Age (years, median)	54 (36–79)	52 (36–79)	57 (43–64)	
Sex				
Female	12 (42.9)	5 (41.7)	7 (43.8)	0.912
Male	16 (57.1)	7 (58.3)	9 (56.2)	
ECOG				
0–1	22 (78.6)	10 (83.3)	12 (75.0)	0.595
2	6 (21.4)	2 (16.7)	4 (25.0)	
Primary tumor site				
GBC	9 (32.1)	3 (25.0)	6 (37.5)	0.483
CCA	19 (67.9)	9 (75.0)	10 (62.5)	
Metastatic site				
Intra-abdominal lymph node	16 (57.1)	6 (50.0)	10 (62.5)	
Liver	14 (50.0)	2 (16.7)	12 (75.0)	0.086
Peritoneum	8 (28.6)	3 (25.0)	5 (31.3)	
Lung	8 (28.6)	6 (50.0)	2 (12.5)	
Others	16 (57.1)	7 (58.3)	9 (56.3)	
Number of metastatic sites				
1–2	13 (46.4)	5 (41.7)	8 (50.0)	0.662
≥ 3 Treatment line	15 (53.6)	7 (58.3)	8 (50.0)	
2	11 (39.3)	5 (41.7)	6 (37.5)	0.823
3–4	17 (60.7)	7 (58.3)	10 (62.5)	

*ECOG* Eastern Cooperative Oncology Group performance status, *GBC* gallbladder cancer, *CCA* Cholangiocarcinoma

**Table 2:** Characteristics of Combination therapy

Characteristic	Total n (%)
Capecitabine	1 (6.3)
S-1	8 (50.0)
Oxaliplatin	1 (6.3)
Irinotecan	2 (12.2)
PD-1 inhibitor	4 (25.0)

PD-1 Programmed cell death-1

### Efficacy

**Fig. 1:**
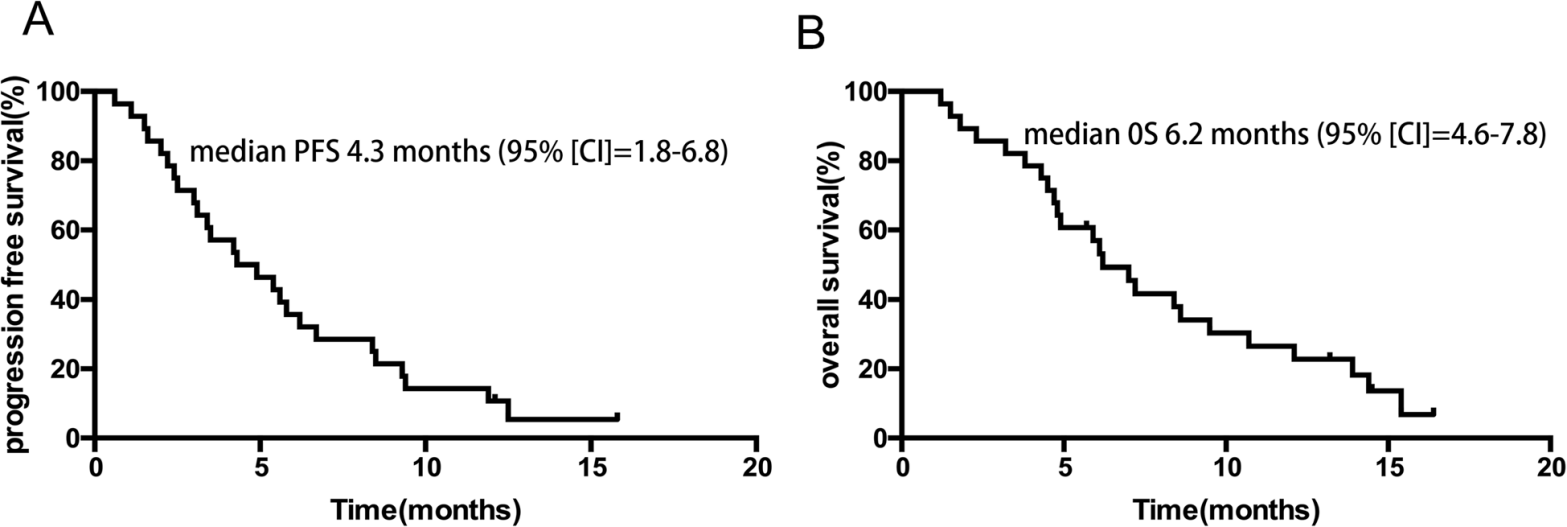
Kaplan–Meier curve of progression-free survival (**a**) and overall survival (**b**)

Treatment efficacy was evaluated in the 28 patients with advanced or recurrent BTC who progressed after prior systemic therapies, CR was not observed. In the general population, 6 patients achieved PR, 15 patients had SD and 7 patients had PD. The ORR and DCR were 21.4% (6/28) and 75.0% (21/28), respectively. In apatinib monotherapy population, 2 patients achieved PR, 8 patients had SD and 2 patients had PD, the ORR and DCR were 16.7% (2/12) and 83.3% (10/12), respectively. In combination therapy population, 4 patients achieved PR, 7 patients had SD and 5 patients had PD, the ORR and DCR were 25.0% (4/16) and 68.8% (11/16), respectively (Table 3). With a median follow-up of 14.8 months (range, 7.1 m to 35.7 m), median PFS and OS in the 28 patients with advanced or recurrent BTC patients were 4.3 months (95% confidence interval [CI] = 1.8–6.8)(Fig. 1a) and 6.2 (95% CI = 4.6–7.8) months (Fig. 1b) respectively. The median PFS in patients who received mono- and combo-regimens was 4.2(95% CI = 2.8–5.6) and 4.9(95% CI = 0.4–9.4) months respectively, and OS was 7.0(95% CI = 4.7–9.2) and 6.1 (95% CI = 3.8–8.4) months respectively.

**Table 3:** Efficacy of apatinib in patients with BTC

Parameter	Total(*n* = 28) n (%)	Monotherapy(*n* = 12) n (%)	Combination therapy(*n* = 16) n (%)	*P*
Best response				0.478
CR	0 (0)	0 (0)	0 (0)	
PR	6 (21.4)	2 (16.7)	4 (25.0)	
SD	15 (53.6)	8 (66.6)	7 (43.8)	
PD	7 (25.0)	2 (16.7)	5 (31.2)	
ORR	6 (21.4)	2 (16.7)	4 (25.0)	0.595
DCR	21 (75.0)	10 (83.3)	11 (68.8)	0.378
Median PFS (months)	4.3	4.2	4.9	0.867
Median OS (months)	6.2	7.0	6.1	0.871

*CR* Complete response, *PR* Partial response, *SD* Stable disease, *PD* Progressive disease, *ORR* Overall response rate, *DCR* Disease control rate, *PFS* Progression free survival, *OS* Overall survival

### Safety

In terms of safety, apatinib treatment was well tolerated, disease progression was the leading cause of discontinuation of treatment, and only 1 patient discontinued treatment due to intolerable toxicity. Most of the adverse events were grade 1–2 in severity, which could be relieved by symptomatic treatment (Table 4). A small number of patients experienced grade 3–4 adverse reactions, including secondary hypertension (2 cases), hand-foot syndrome (1 case) and neutropenia (1 case). No unexpected side effects or treatment-related death were observed. Dose reduction occurred in two patients adjusting the dosage from 500 to 250 mg due to adverse events in apatinib monotherapy population. In apatinib monotherapy population, the most common adverse events were secondary hypertension(*n* = 5; 41.7%), hand and foot syndrome(*n* = 3; 25.0%), fatigue(*n* = 3; 25.0%) and proteinuria(*n* = 2; 16.7%). In combination therapy population, the most common adverse events were neutropenia(*n* = 7; 43.8%), nausea(*n* = 4; 25.0%), secondary hypertension(*n* = 3; 18.8%), hand and foot syndrome(n = 3; 18.8%).

**Table 4:** Treatment-Related Toxicities

Adverse Event	Grade1 n(%)	Grade2 n(%)	Grade3 n(%)	Grade4 n(%)	Total n(%)
Monotherapy group(*n* = 12)					
Secondary hypertension	1 (8.3)	2 (16.7)	1 (8.3)	1 (8.3)	5 (41.7)
Hand-foot syndrome	1 (8.3)	1 (8.3)	1 (8.3)	0	3 (25.0)
Proteinuria	2 (16.7)	0	0	0	2 (16.7)
Fatigue	1 (8.3)	2 (16.7)	0	0	3 (25.0)
Diarrhea	1 (8.3)	0	0	0	1 (8.3)
Combination group(*n* = 16)					
Secondary hypertension	2 (12.5)	1 (6.3)	0	0	3 (18.8)
Hand-foot syndrome	1 (6.3)	2 (12.5)	0	0	3 (18.8)
Proteinuria	2 (12.5)	0	0	0	2 (12.5)
Nausea	2 (12.5)	2 (12.5)	0	0	4 (25.0)
Neutropenia	5 (31.3)	1 (6.3)	1 (6.3)	0	7 (43.8)

## Discussion

BTC is a rare but highly malignant tumor. 60–70% of new BTC cases are diagnosed as advanced stage [13, 14]. Patients of advanced BTC have a very poor prognosis with a median OS less than 12 months [14]. Prospective randomized control studies have shown that systemic chemotherapy can prolong the survival of patients with advanced BTC compared with optimal supportive care [15].

Based on the results of a phase III randomized controlled ABC-02 study, gemcitabine and cisplatin are recommended as the first-line standard treatment regimen for advanced, which can reduce the OS of patients with advanced BTC from 8.1 Month increased to 11. Seven months [16]. In the meantime, gemcitabine combined with S-1 was also recommended as first-line treatment options for advanced BTC [17, 18]. However, there are no clear second-line treatment options for those with disease progression after first-line treatment. Summary of first-line treatment in BTC and in advanced BTC are displayed in Table S1 and Table S2. New treatment strategies are urgently needed.

Currently, cancer therapy has entered the era of molecular therapy, and targeted therapy based on genetic changes has become an effective strategy for cancer [19]. Numerous studies have demonstrated the landscape of molecular mutations in BTC, including EGFR pathway, HER2 (ERBB2), VEGF pathway, PI3K/mTOR signaling cascade, FGFR pathway, IDH1, MEK pathway and etc. [20–23]. As a tyrosine kinase inhibitor that selectively inhibits the vascular endothelial growth factor receptor-2 (VEGFR2), apatinib has showed good clinical efficacy in a variety of solid tumors. Previous study demonstrated that apatinib inhibits VEGF-mediated cell migration and invasion of CCA cell lines [12], and apatinib promotes apoptosis in intrahepatic cholangiocarcinoma [24], which highlight the potential clinical utility of apatinib in BTC. However, clinical use of apatinib in BTC is rarely reported. Only one case report investigated the clinical efficacy and safety of apatinib in advanced or recurrent BTC [25].

In our present study, treatment efficacy of apatinib was evaluated in the 28 patients with advanced or recurrent BTC who progressed after prior systemic therapies. Although CR was not observed, the ORR and DCR reached to 21.4 and 75.0% respectively, median PFS and OS were 4.3 month and 6.2 months. There are very few studies on standard second-line treatment regimens after first-line chemotherapy failure in BTC. In the phase III randomized controlled ABC-06 study, oxaliplatin combined with 5-FU (mFOLFOX) solution second-line chemotherapy increased OS from 5.3 months to 6.2 months, with a median PFS of 4.0 months. In another randomized phase II study of second-line XELIRI regimen versus irinotecan monotherapy in BTC, median PFS were 3.7 and 2.4 months respectively [26]. PFS and OS survival in our present study was superior. It is noteworthy that, approximately 60% patients received apatinib as third- or fourth-line treatment, but still got a good response and improved survival.

Apatinib targeted therapeutics showed efficacy both in apatinib monotherapy and combination therapy patients. In combination therapy population, 3 patients received apatinib plus PD-1 inhibitor, of which one patient achieved PR, and the other 2 patients had SD. Our study suggested that compared with traditional pure cytotoxic drugs, targeted therapy has unique advantages and application prospects. For patients with advanced BTC, targeted therapy or targeted therapy combined with chemotherapy and immunotherapy may be new treatment strategies, and more clinical researches should be conducted [27].

Advanced or recurrent BTC is incurable, and its treatment is palliative, with the goal of extending survival. Gentle, effective and concerned about the quality of life are the basic principles of treatment. Therefore, treatment-related toxicity is also a major evaluation indicator. Commonly reported apatinib-related AEs included secondary hypertension, hand and foot syndrome, fatigue and proteinuria, a similar safety profile was observed in the present study. Most of the adverse events were grade 1–2 in severity, and no unexpected side effects or treatment-related death were observed. Dose reduction occurred in only two patients adjusting the dosage from 500 to 250 mg due to adverse events in apatinib monotherapy population. In combination therapy population, the above incidence of apatinib-related adverse reactions is lower, but the incidence of hematological toxicity is relatively higher due to utilization of chemotherapy. In general, apatinib treatment was well tolerated.

Our study has some limitations, because it is a retrospective study obtained from a single center, and the number of cases enrolled is not very large. However, the efficacy that median PFS in the 28 patients with advanced or recurrent BTC patients who had undergone prior systemic therapies reached to 4.3 months is still encouraging. Thus, large randomized controlled trials from multi-center are needed to confirm the clinical value of apatinib in advanced or recurrent BTC.

## Conclusions

In conclusion, our present study evaluated the efficacy and safety of apatinib in advanced or recurrent BTC, a median PFS of 4.3 months was obtained with well tolerated toxicity. These promising results support apatinib therapy as a feasible therapeutic strategy in advanced or recurrent BTC.

## Supplementary Information

**Additional file 1.**

**Additional file 2.**

## Data Availability

The datasets used and analyzed during the current study are available from the corresponding author on reasonable request.

## Abbreviations

BTC: Biliary tract cancer
PFS: Progression free survival
OS: Overall survival
ORR: Objective response rate
DCR: Disease control rate
GBCs: Gallbladder cancers
CCAs: Cholangiocarcinomas,
iCCAs: Intrahepatic cholangiocarcinomas
eCCAs: Extrahepatic cholangiocarcinomas
CR: Complete response
PR: Partial response
SD: Stable disease
PD: Progressive disease
AEs: Adverse events
