# Supplementary material for: The efficacy and safety of apatinib treatment for patients with advanced or recurrent biliary tract cancer: a retrospective study
Source: BMC Cancer. 2021 Feb 23;21:189. doi: 10.1186/s12885-021-07907-4 (PMC7903638; doi:10.1186/s12885-021-07907-4)
Supplement: Supplementary file 1 — Additional file 1. [file 12885_2021_7907_MOESM1_ESM.docx]

**Table S1 Summary of first-line treatment in BTC**

| Regimen | PFS | OS |
| --- | --- | --- |
| GemCis vs. Gem alone | 8.0 vs. 5.0 months | 11.7 vs. 8.1 months |
| GAP | 11.8 months | 19.2 months |
| FOLFIRINOXm | n.a | n.a |
| Nal-IRI | n.a | n.a |
| GEM | 5.0 months | 8.3 months |
| GEM + CDDP | 8.0 months | 11.7 months |
| GEM + S-1 | 7.1 months | 12.5 months |
| GEM + L-OHP | 4.2 months | 9.5 months |
| GEM + sorafenib | 3.0 months | 8.0 months |
| Vandetanib | 3.5 months | 7.5 months |
