# Supplementary material for: The efficacy and safety of apatinib treatment for patients with advanced or recurrent biliary tract cancer: a retrospective study
Source: BMC Cancer. 2021 Feb 23;21:189. doi: 10.1186/s12885-021-07907-4 (PMC7903638; doi:10.1186/s12885-021-07907-4)
Supplement: Supplementary file 2 — Additional file 2. [file 12885_2021_7907_MOESM2_ESM.docx]

**Table S2 Summary of treatment in advanced BTC**

| Regimen | PFS | OS |
| --- | --- | --- |
| ASC | n.a | 5.3 months |
| ASC+ mFOLFOX6 | n.a | 6.2 months |
| XELIRI VS. IRI | 3.7 vs. 2.4 months | 10.1 vs. 7.3 months  DCR: 63.3% vs. 50.0% |
| FOLFIRI + bevacizumab | 8.0 months | 20.0 months |
| FOLFIRI | 3.2 months | 21.9 months |
| FTD/TPI | 3.8 months | 6.1 months |
| Lapatinib | 1.8 months | 5.2 months |
| Everolimus | 3.2 months | 7.7 months |
| Cabozantinib | 1.8 months | 5.2 months |
| Erlotinib | 2.6 months | 7.5 months |
